# Pathological beta power increase in the subthalamic nucleus is absent in essential tremor

**DOI:** 10.1093/braincomms/fcaf297

**Published:** 2025-08-14

**Authors:** Halen Baker Erdman, Hagai Bergman, Juan F Leon, Sami Heymann, Yara Atamna, Muneer Abu Snineh, Omer Zarchi, Idit Tamir, Zvi Israel

**Affiliations:** Department of Medical Neurobiology, Hebrew University of Jerusalem, Jerusalem 91120, Israel; Department of Medical Neurobiology, Hebrew University of Jerusalem, Jerusalem 91120, Israel; Edmond and Lily Safra Center for Brain Sciences, Hebrew University of Jerusalem, Jerusalem 91904, Israel; Department of Neurosurgery, Hadassah Medical Center, Jerusalem 91120, Israel; Department of Neurosurgery, Hadassah Medical Center, Jerusalem 91120, Israel; Department of Neurosurgery, Hadassah Medical Center, Jerusalem 91120, Israel; Department of Neurosurgery, Hadassah Medical Center, Jerusalem 91120, Israel; Department of Neurology, Hadassah Medical Center, Jerusalem 91120, Israel; Intraoperative Neurophysiology Unit, Rabin Medical Center, Petah Tikva 49100, Israel; Department of Neurosurgery, Rabin Medical Center, Petah Tikva 49100, Israel; Department of Neurosurgery, Hadassah Medical Center, Jerusalem 91120, Israel

**Keywords:** beta oscillations, posterior subthalamic area, dual electrode, motor disorder, tremor

## Abstract

Parkinson’s disease and essential tremor are common movement disorders characterized by distinct motor symptoms. Deep brain stimulation targeting the subthalamic nucleus has shown efficacy in managing Parkinson’s disease symptoms, whereas the posterior subthalamic area is an emerging target for essential tremor. This analytical cross-sectional study investigates the electrophysiological activity of the subthalamic nucleus in Parkinson’s disease and essential tremor patients from deep brain stimulation surgeries to understand the underlying neural oscillatory mechanisms. Microelectrode recordings during deep brain stimulation surgery from 35 Parkinson’s disease patients targeting the subthalamic nucleus and 21 essential tremor patients simultaneously targeting the posterior subthalamic area and subthalamic nucleus using a novel dual electrode technique were analysed for the main analysis. Additionally, subthalamic nucleus data from a subgroup of 12 Parkinson’s disease patients was compared with seven essential tremor patients who were matched based on the *y*-coordinate of the electrode. A final comparison was made between a third subgroup of nine Parkinson’s disease patients with satisfactory subthalamic nucleus recordings in the posterior BenGun location and 21 essential tremor patients. Recordings were collected from two medical centres with a common electrophysiology team. Root mean square and spectral analysis were employed as well as statistical analysis of demographic and recorded subthalamic nucleus anatomical dimensions. Relative dimensions of subthalamic nucleus physiological regions did not differ between the main groups. The motor subregion of the subthalamic nucleus in Parkinson’s disease patients exhibited significantly increased beta frequency power (13–30 Hz). Conversely, essential tremor patients did not show this increase, suggesting distinct pathophysiological mechanisms. Additionally, the subthalamic nucleus spiking activity, as measured by RMS analysis, was higher in Parkinson’s disease patients. *y*-coordinate matched, and posterior subthalamic nucleus Parkinson’s disease patient comparisons confirmed the higher beta frequency power in Parkinson’s disease patients only. These findings underscore the different neural dynamics between Parkinson’s disease and essential tremor. They highlight the role of beta oscillations in Parkinson’s disease’s motor symptoms and raise questions about the absence of beta oscillations in essential tremor, whether it reflects a normal lack of beta activity or an active suppression of a normal beta stop signal.

## Teaser

Baker Erdman *et al*. investigate the electrophysiological activity of the subthalamic nucleus in Parkinson’s disease and essential tremor patients from deep brain stimulation surgeries to understand the underlying neural oscillatory mechanisms. They find that the subthalamic nucleus in Parkinson’s disease patients exhibits significantly increased beta frequency power, suggesting distinct pathophysiological mechanisms.

## Graphical Abstract

**Graphical Abstract:**
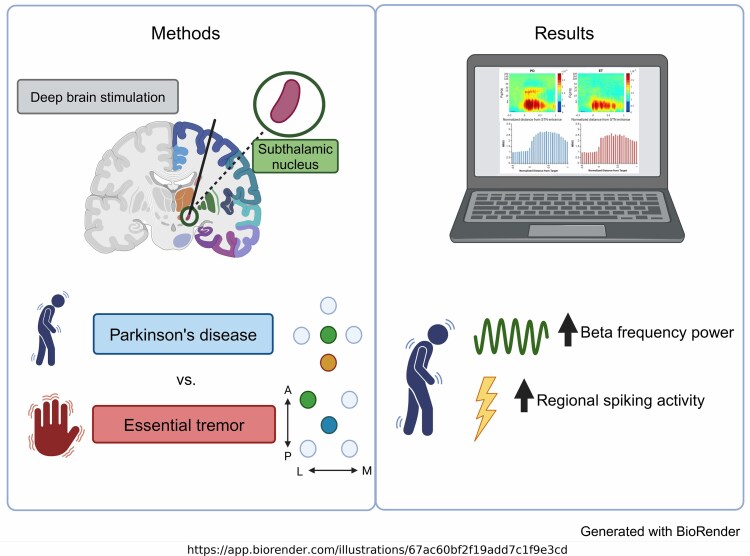

## Introduction

Parkinson’s disease and essential tremor are among the most prevalent movement disorders, characterized by distinct motor symptoms that impact patients’ quality of life.^1,2^ Deep brain stimulation (DBS) has emerged as an effective therapeutic approach for managing these conditions, targeting specific subcortical nuclei to alleviate motor symptoms. The subthalamic nucleus (STN), a crucial component of the basal ganglia circuitry, has gained significant attention due to its role in motor control and its modulation through DBS.^3^ Notably, many studies have highlighted the importance of investigating specific subregions within the STN to better understand the pathophysiological mechanisms underlying these disorders and the effects of DBS.^4-8^

Traditionally, the ventral intermediate nucleus (VIM) of the thalamus has been the primary target for DBS in essential tremor, yielding notable tremor reduction outcomes.^9^ However, many patients have a narrow therapeutic window and a deterioration of therapeutic effects over time.^10^ The advent of refined neuroimaging techniques and functional neuroanatomy studies has revealed an alternative target that holds promise in refining essential tremor treatment outcomes: the posterior subthalamic area (PSA). Both VIM and PSA DBS target the cerebral-rubro-thalamic (CRT) tract,^11^ so their relative effects depend on the stimulation of the CRT tract. However, it seems that the PSA might provide a better outcome than the VIM.^12^

Microelectrode recordings (MERs) obtained during DBS surgery offer valuable insights into the oscillatory dynamics of the STN, shedding light on the distinct pathophysiological mechanisms in Parkinson’s disease and essential tremor. Notably, previous studies have demonstrated increased beta frequency (13–30 Hz) power within the motor subregion of the STN in Parkinson’s disease patients,^13-16^ supporting the idea that excessive beta oscillations contribute to the motor symptoms observed in this disorder. However, the electrophysiological signatures within the motor subregion of the STN in essential tremor patients have remained unexplored. To address this gap in knowledge, we conducted a comparative study utilizing MERs obtained during DBS surgery. Importantly, our study design involved targeting the STN in Parkinson’s disease patients and simultaneously the PSA and STN in essential tremor patients using a dual-microelectrode technique. Given the limited electrophysiological signatures in the PSA, this approach allowed us to capture relevant neural activity while concurrently increasing the accuracy of lead placement in the PSA. In this study, we aimed to investigate whether the motor subregion of the STN exhibits a similar increase in beta frequency power in essential tremor patients as observed in Parkinson’s disease patients.

## Materials and methods

### Participants

Thirty-five patients with idiopathic Parkinson’s disease (62 STN trajectories) and 21 patients with essential tremor (31 STN trajectories) who were due to undergo DBS targeting the STN and the PSA, respectively, were included in this study. For the main analysis, all patients from both groups were included. To overcome potential differences in the *y*-coordinates due to differing main targets (STN versus PSA), a set of patients was selected with the closest *y*-coordinates. The *y*-matched Parkinson’s disease group consisted of 12 patients (15 trajectories), and the *y*-matched essential tremor group consisted of 7 patients (10 trajectories). Again, to overcome potential differences in location within the STN, a third group of Parkinson’s disease patients (9 patients, 15 trajectories) was selected, which showed satisfactory recordings from the electrode located in the posterior BenGun position. This posterior Parkinson’s disease group was then compared with the full set of essential tremor patients. Thus, three group comparisons were made: (i) all Parkinson’s disease versus all essential tremor patients; (ii) *y*-coordinate matched Parkinson’s disease versus essential tremor patients; and (iii) posterior electrode Parkinson’s disease group versus all essential tremor patients. Available data were obtained from Hadassah Medical Center and Rabin Medical Center with surgery dates from 2020 to 2023.

### Study design

This study is an analytical cross-sectional study on existing data from standard-of-care DBS surgeries. All patients were given their diagnosis by movement disorder specialist. Surgical target (STN/PSA) and coordinates were determined based on patient disease by the neurosurgeon without relation to this study. The primary outcomes are the beta frequency power and the power of the regional spiking activity within the STN. The secondary outcomes of this study are electrophysiological recording length of the STN, delta, theta, alpha and gamma frequency band power analysis, as well as analysis of the main outcomes in the *y*-coordinate matched and posterior STN groups.

### Surgery protocol

**Figure 1:**
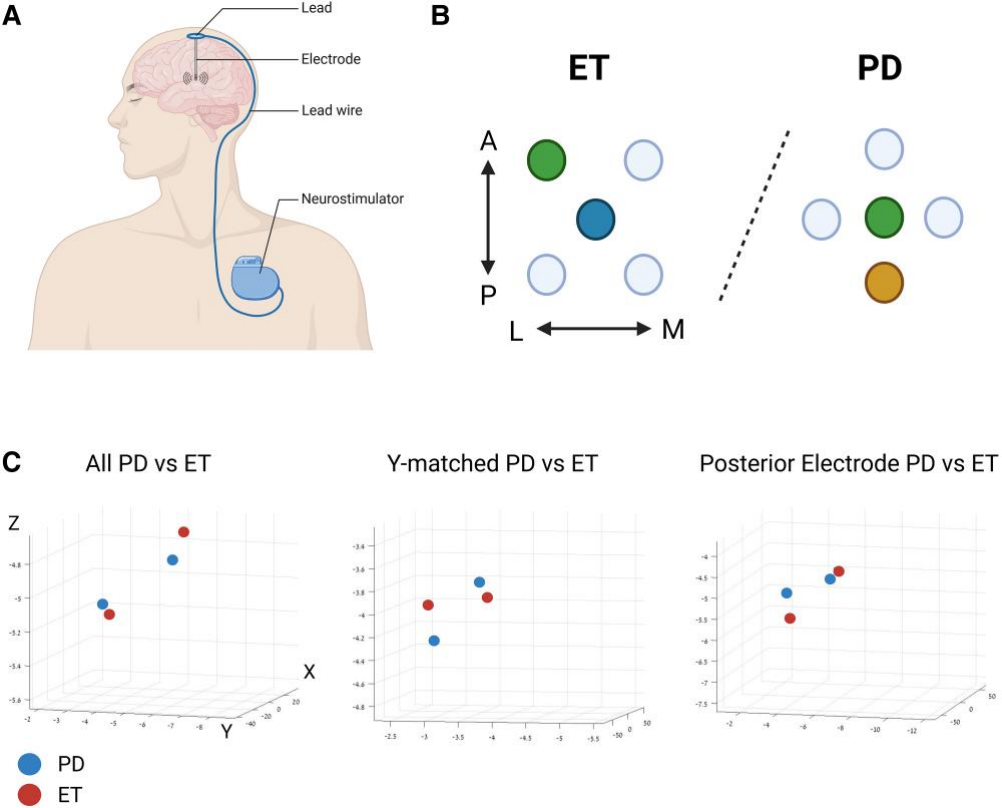
**Surgical technique and average electrode location.** (**A**) Schematic of the elements implanted during DBS surgery. (**B**) BenGun setup for DBS targeting the PSA and STN for the treatment of essential tremor (left) and targeting the STN only for the treatment of Parkinson’s disease (right). Green circle represents the BenGun position that targets the STN, blue circle represents PSA and gold represents the posterior STN BenGun location. (**C**) The individual trajectory and average *x*- (absolute value), *y*- and *z*-coordinates for each group comparison. Darker blue dot with ‘x’ represents the average Parkinson’s disease group, lighter blue dots represent individual trajectories in the Parkinson’s disease group, darker red dot with ‘x’ represents the average essential tremor group and lighter red dots represent individual trajectories in the essential tremor group in each graph. Created in BioRender. [Halen Erdman]. (2025) https://app.biorender.com/illustrations/6694c2d5ad9ae26508fd62b5.

A schematic of the surgical protocol is shown in Fig. 1A. All patients stopped taking medication 12 h prior to surgery. A stereotactic frame (CRW, Integra Lifesciences, Princeton, NJ) was secured onto the patient’s head using local anaesthesia (lidocaine 2% and bupivacaine 0.5%, approximately 20 cc) and mild sedation (2 mg IV midazolam). Subsequently, a head computed tomography (CT) scan was performed with a fiducial box attached to the stereotactic frame. Fusion of the CT and MRI images was then carried out, and the frame coordinates were extracted from the navigation system (Medtronic Stealth 8). STN targets for Parkinson’s disease patients were defined in preoperative planning in the posterior-ventral third of the STN, 1 mm from the medial border of the nucleus. PSA targets were defined using Claudio Pollo’s method.^17^ Briefly, in the T2 MRI axial section, a horizontal line was created across the equator of the red nucleus at its maximum diameter. A second line, defining the axis of the STN, was drawn from its most anteromedial to the most posterolateral point. A third line, perpendicular to the STN axis, intersected the first horizontal line. The target point was then chosen in the intersection of the first and third lines, or even slightly posterior, positioned midway between the lateral border of the red nucleus and the medial border of the STN. Trajectory angles were set according to the cortical, ventricle and blood vessel anatomy. The patient was transferred to the operating room and positioned in a supine manner, with the frame securely fixed to the operating table. Anaesthesiologists were encouraged to avoid using beta-blockers in both groups of patients if possible, and hypertension was first treated by calcium blockers. Patient monitoring adhered to the guidelines of the American Society of Anesthesiologists (ASA).

The exact sedation regimen varied according to patient needs, but in some patients, prior to skin incision and drilling of the burr-hole, propofol was administered and was utilized to maintain the patient at a moderate level of sedation as per the ASA guidelines. Propofol was stopped upon completion of the burr-hole drilling, approximately 5–20 min before the initiation of MER. In other patients, no sedation was given prior to MER. A subset of essential tremor patients was then administered low-dose ketamine continuous infusion to a level of conscious sedation until the MER was completed. All other patients were not given any sedative for the remainder of the MER period. As for the STN of Parkinson’s disease,^18,19^ we did not find any differences in STN physiological properties with and without ketamine, and the two groups are merged for the below analysis. After burr-hole drilling, the lead fixation device was securely attached to the skull, and the dura, arachnoid and pia matter were coagulated and incised sharply. Subsequently, two guide tubes (in some of the Parkinson’s disease patients, we used only one guide tube) were lowered to a depth of 15 or 25 mm above the predetermined target through a BenGun which holds the guide tubes in place. The BenGun has five channels, central and four lateral channels located 2 mm from the central channel in a + or x configuration, as illustrated in Fig. 1B. In the case of those patients where only one guide tube was used, it was lowered through the central BenGun position. NeuroProbe microelectrodes from Alpha Omega Engineering (Ziporit, Israel) were then introduced through the guide tubes to a starting recording depth of 10 mm above the target.

In both the Parkinson’s disease and essential tremor groups, identification of the STN during MER was conducted by an expert electrophysiologist, with assistance from the hidden Markov model STN detection programme (HaGuide, Alpha Omega Engineering, Ziporit, Israel), as described in previous studies.^20-22^ The STN identification process involved outlining the dorso-lateral and ventro-medial borders of the STN and demarcating its motor and non-motor subdomains. In some patients, an intraoperative CT scan (O-Arm, Medtronic) was performed for final lead validation. Otherwise, postoperative CT was performed 1–2 months after the surgery.

### Posterior subthalamic area targeting

In the essential tremor group, the PSA was the intended target for the final implantation of the lead. A dual electrode technique, in which one microelectrode is lowered in the central BenGun position and targets the PSA and one microelectrode is lowered in the anterior lateral BenGun position (2 mm oblique distance between microelectrodes) and targets the STN, was used. Using this technique, positive identification of the STN in the anterior lateral position indicates the microelectrode in the central position was on target in the PSA (Fig. 1B). This technique harnesses the robust and characteristic electrophysiological signature of the STN in order to indirectly target the relatively electrophysiologically silent PSA and gives us insight into the STN of essential tremor patients.

Following STN/PSA identification, bipolar cathodal-first stimulation was performed from the macro contact of the NeuroProbe. In the Parkinson’s disease group, the stimulation was performed with the macro contact located at the bottom of the motor subregion of the STN. In the essential tremor group, the stimulation was performed with the macro contact of the central (PSA) electrode at the level of 0 mm above the target if the ventral border of the STN as determined by the intraoperative electrophysiology was at or above 0, or at the level of the ventral border of the STN if it was below 0 mm above target. The average final electrode location for each group is shown in Fig. 1C and detailed in Table 1.

**Table 1:** Patient demographics and electrode coordinates

	Parkinson’s disease (all)	Essential tremor (all)	Parkinson's disease (*y*-matched)	Essential tremor (*y*-matched)	Parkinson’s disease (posterior)
Patient demographics					
# of patients	35	21	12	7	9
Age, years	64.6 (10.4)	69.9 (10.0)	62.1 (5.7)	69.3 (3.7)	62.7 (8.7)
Sex, M:F	23:12	6:15	9:3	2:5	4:5
STN coordinates (relative to MCP, mm)					
*x* (L/R)	−11.0 (4.5)/11.7 (1.0)	−10 (1.0)/9.7 (0.9)	−12.6 (1.3)/12.4 (0.9)	−11.6 (0.6)/12.2 (0.8)	−11.8 (0.5)/12.1 (0.8)
*y* (L/R)	−3.3 (0.8)/−2.9 (0.9)	−5.8 (0.8)/−5.6 (0.9)	−2.8 (0.6)/−2.6 (0.2)	−3.6 (0.5)/−3.4 (1.2)	−3.6 (0.6)/−2.9 (0.8)
*z* (L/R)	−5.1 (1.9)/−5.2 (0.7)	−4.8 (1.0)/−4.7 (0.9)	−4.3 (1)/−4 (0.1)	−3.9 (0.5)/−3.8 (1.3)	−5.7 (0.7)/−5.3 (0.8)
Arc (L/R)	21.7 (2.0)/21.0 (2.7)	23.5 (3.2)/24.5 (2.6)			20.7 (1.7)/20.4 (2.6)
Ring (L/R)	61.2 (4.5)/60.3 (4.2)	60.1 (6.4)/60.0 (7.3)			64.0 (4.5)/63.5 (3.4)

STD shown in parentheses. Arc—angle from the sagittal plane. Ring—angle from the axial plane.

### Electrophysiological data collection

One or two microelectrodes were positioned 10 mm above the planned target, with recordings taken at 0.4 mm intervals before entering the STN and at 0.1 mm intervals within the STN using the NeuroOmega navigation system (Alpha Omega Engineering, Ziporit, Israel). At each site, recordings began after a 2 s stabilization period and lasted for 4 s, totalling 6 s per site. The entrance to the STN was identified by an increase in background noise from baseline levels, high-density firing, and an increase in delta, theta and beta frequency bands. This was visualized using the real-time analysis and automatic algorithm of the HaGuide (Alpha Omega Engineering, Ziporit, Israel). An increase in the power of the beta and lower frequency bands was used to identify the dorsolateral oscillating region (DLOR, motor domain) of the STN. The ventromedial non-oscillating region (VMNR, non-motor domain) of the STN was characterized by broad frequency gamma activity and covered the remaining part of the trajectory. The exit from the STN was determined by a decrease in background noise level, the end of high-density firing, or the appearance of electrophysiological signs of the substantia nigra.^20^ All patients were conscious during electrophysiological recording. Most patients were given only saline during this phase; however, some patients received low-dose ketamine resulting in conscious sedation (eyes open and responsive).

### Electrophysiological data analysis

Electrophysiological data analysis was conducted offline as described in Baker Erdman *et al*.^18^ Specifically, spiking activity (filtered to a 300–6000 Hz band-pass) was extracted using the NeuroOmega navigation system (Alpha Omega Engineering, Ziporit, Israel). For each recording site, the power spectrum density (PSD) and root mean square (RMS) were computed. The RMS was normalized to the RMS level of the first five to six sites, taken 0.1 mm apart. These sites are located 10 mm above the planned target while still outside the STN, typically in the internal capsule. The PSD was calculated from rectified data (using the absolute operator) and normalized to the RMS (total power in the recorded frequency range). This way, the PSD provided detailed information about the distribution of the power of the spiking activity envelope at discrete frequency bins (1/3 Hz resolution) as a fraction of the total power at a site, making it independent of total power changes. STN borders and subdomains were determined using a previously published algorithm,^21^ and corrected, if necessary, by an expert electrophysiologist (H.B.E.).

For the main analysis including all Parkinson’s disease and essential tremor patients, and for the *y*-coordinate matched group analysis, the trajectory that was determined to have the best STN recording (usually the one chosen for implantation in the Parkinson'’ disease patients and the anterior lateral trajectory in essential tremor patients) was included in the analysis. For the posterior electrode Parkinson’s disease group analysis, trajectories with satisfactory STN recordings in the electrode located in the posterior BenGun position were selected for analysis. The recorded STN length was extracted from trajectory recordings for each patient, and the length was normalized to a STN length of 1 to enable group analysis. For each trajectory, a segment before STN entrance equal to half the length of the STN from that trajectory was included in the normalized visualizations. PSD and normalized RMS (NRMS) for the STN of individual patients were grouped and averaged to create an average PSD and NRMS for each patient group.

Beta band data were extracted using a four-pole band-pass filter from 13 to 30 Hz. The beta power within the DLOR compared to the beta power outside the STN was calculated as the beta ratio for each trajectory and then analysed across groups. Notably, this frequency range is broader than the typical beta range of a typical patient and, therefore, provides a lower estimate for the increase in beta activity for Parkinson’s disease patients. The peak value and area under the curve (AUC) of the NRMS were computed for each trajectory and then evaluated across groups. All analysis was performed using custom Matlab (v2017a) scripts.

### Statistical analysis

After the patient data were gathered and grouped, statistical null assumptions were tested. Parametric tests were applied to data with a normal distribution (electrophysiological data parameters), while non-parametric tests were used for data that were not normally distributed (demographic patient data). Results are presented as mean ± standard error mean (SEM) for continuous normally distributed variables, average ± standard deviation for non-normally distributed data and number (%) for categorical variables. Group comparisons were made using ANOVA or the Kruskal–Wallis test. A *P*-value of less than 0.05 was considered significant.

## Ethics statement

Due to the use of available data for this study, informed consent was waived for patients from Hadassah Medical Center. Informed consent was obtained prior to surgery for the nine essential tremor patients from Rabin Medical Center. Helsinki approval was obtained from Hadassah Medical Center (0168-10-HMO) and Rabin Medical Center (0004-20-RMC).

## Results

Available patient demographic data are shown in Table 1. No age differences were found between groups (*P* = 0.065). However, there was a significant difference in sex between groups (*P* = 0.01). Parkinson’s disease patients had an average preoperative UPDRS score of 40.5 off medication and 20.1 on medication. TETRAS scores were unavailable for essential tremor patients.

### Relative subthalamic nucleus subregion lengths do not differ between the Parkinson’s disease and the essential tremor groups

**Figure 2:**
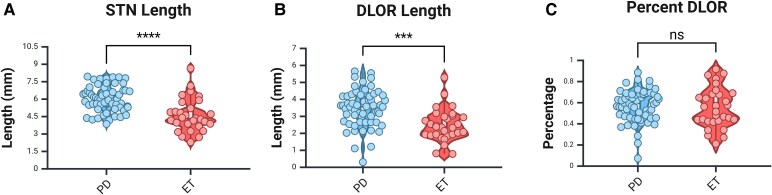
**STN anatomical dimensions.** (**A**) Electrophysiologically recorded STN length differed between essential tremor and Parkinson’s disease groups. Unpaired *t*-test, *P* < 0.001, *t* = 5.29, *n* = 31 for essential tremor, *n* = 62 for Parkinson’s disease. (**B**) Electrophysiologically recorded DLOR length differed between essential tremor and Parkinson’s disease groups. Unpaired *t*-test, *P* < 0.001, *t* = 4.19, *n* = 31 for essential tremor, *n* = 62 for Parkinson’s disease. (**C**) Percentage DLOR (DLOR/total length) did not differ between groups. Unpaired *t*-test, *P* = 0.63, *t* = 0.48, *n* = 31 for essential tremor, *n* = 62 for Parkinson’s disease. Blue represents Parkinson’s disease group, and red represents essential tremor group. Dots represent individual trajectories, white box represents the interquartile range, solid horizontal bar within the white box shows the median, whiskers show minimum and maximum and width of the violin shows density of data points. Created in BioRender. [Halen Erdman]. (2025) https://app.biorender.com/illustrations/6683e8db5a2c9c1d8901d96c.

Total STN length was calculated for all Parkinson’s disease and essential tremor patients and averaged 6 and 4.6 mm, respectively. Likewise, the recorded length of the motor subregion of the STN (DLOR) was computed and averaged 3.5 mm in the Parkinson’s disease group and 2.5 mm in the essential tremor group. While the total and motor subregion lengths did differ significantly (Fig. 2A, *P* < 0.001; Fig. 2B, *P* < 0.001), it is important to note that this could be due to the trajectory differences that were necessary to reach their respective final targets (STN/PSA) and causing the recording from the essential tremor patients to be from a more posterior part of the STN. As such, we calculated the percentage of the recorded STN length that was designated as DLOR out of the total STN length for each trajectory and found no significant difference between the two groups (Fig. 2C, *P* = 0.63). The average per cent DLOR in the Parkinson’s disease group was 57.1% and 55.4% in the essential tremor group.

### Regional spiking activity is attenuated in essential tremor patients

**Figure 3:**
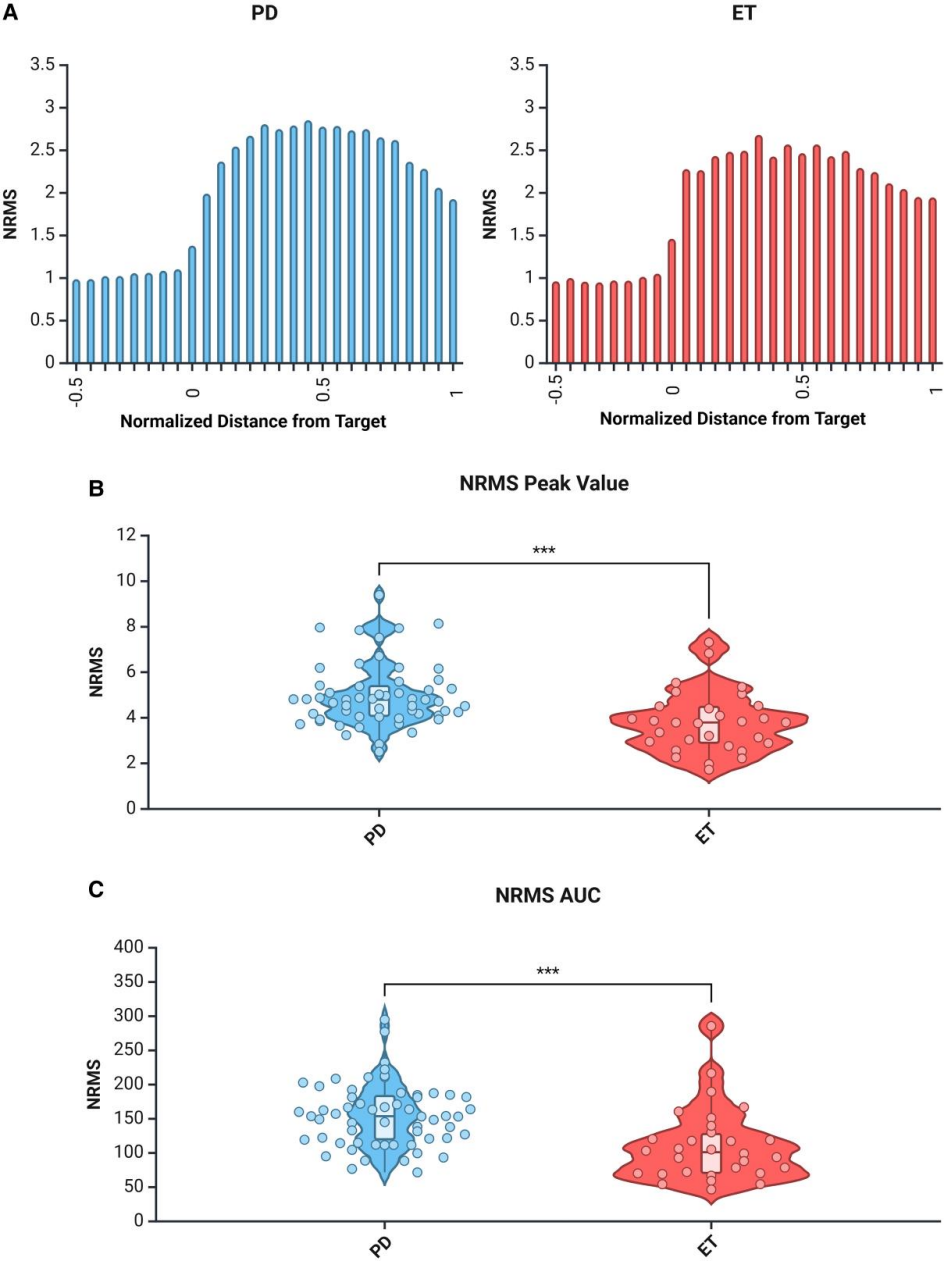
**Regional spiking activity is increased in the STN of Parkinson’s disease patients.** (**A**) Group-averaged NRMS analysis along the trajectory in the full Parkinson’s disease group (left, blue) and full essential tremor group (right, red). Zero represents the entrance to the STN and one represents the exit. (**B**) Peak NRMS values were increased in Parkinson’s disease patients in comparison with essential tremor patients. Unpaired *t*-test, *P* < 0.001, *t* = 3.77, *n* = 31 for essential tremor, *n* = 62 for Parkinson’s disease. (**C**) AUC of the NRMS was increased in Parkinson’s disease patients. Unpaired *t*-test, *P* < 0.001, *t* = 4.06, *n* = 31 for essential tremor, *n* = 62 for Parkinson’s disease. In **B** and **C**, Parkinson’s disease group is shown in blue and essential tremor group is shown in red, dots represent individual trajectories, white box represents the interquartile range, solid horizontal bar within the white box shows the median, whiskers show minimum and maximum and width of the violin shows density of data points. Created in BioRender. [Halen Erdman]. (2025) https://app.biorender.com/illustrations/668a3b3cc7f175be5d6031ce.

To understand the overall activity level of the nucleus, we examined the NRMS of the spiking activity (300–6000 Hz) along the trajectories. Both groups showed the expected increase in the NRMS as the STN was entered and the decrease at its exit (Fig. 3A). However, when quantifying the peak of the NRMS (Fig. 3B) and AUC (Fig. 3C), we found that the regional spiking activity was significantly attenuated in the essential tremor group when compared to the Parkinson’s disease group (*P* < 0.001 for peak NRMS, *P* < 0.001 for AUC). This is somewhat to be expected as the STN is thought to be overactive in Parkinson’s disease.^23,24^

### Beta frequency power is distinctively increased in Parkinson’s disease patients

**Figure 4:**
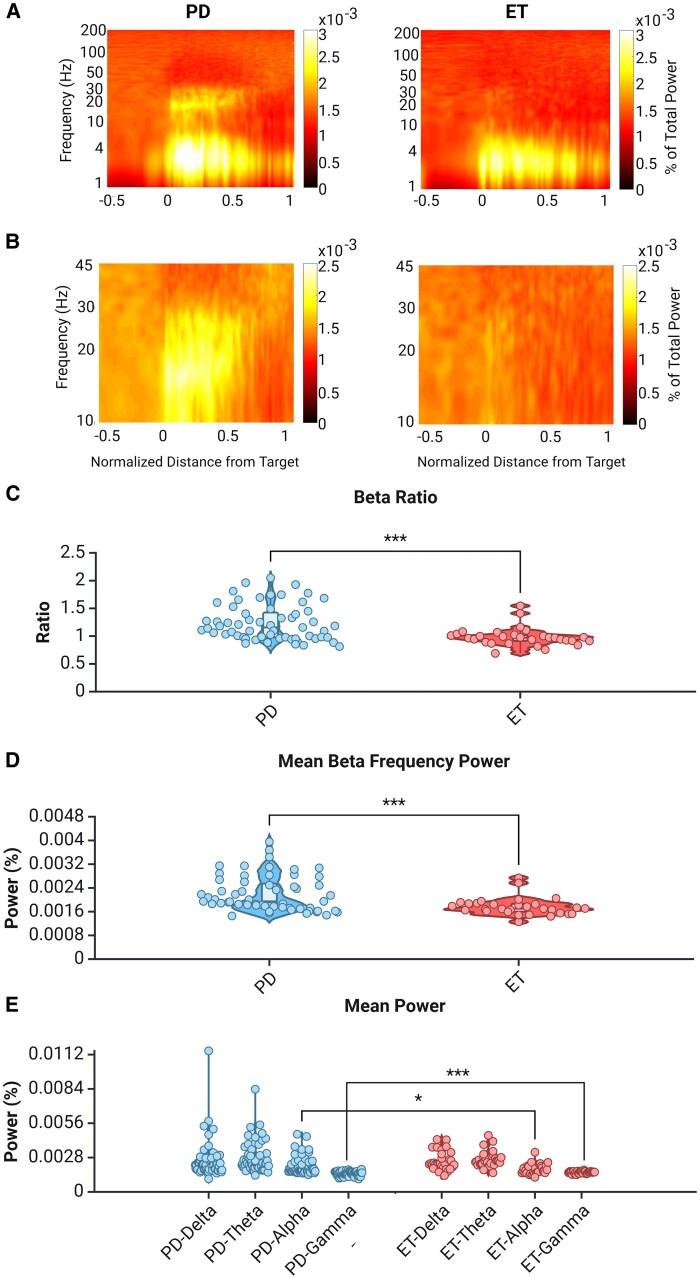
**Beta band frequency power is increased in the motor subregion of Parkinson’s disease patients.** (**A**) Group-averaged spectrograms for the Parkinson's disease group (left) and essential tremor group (right) for a frequency range of 1–200 Hz. (**B**) Recalculated and zoomed-in group-averaged spectrograms for the Parkinson's disease group (left) and essential tremor group (right) to better see the beta band (13–30 Hz) for a frequency range of 10–45 Hz. (**C**) The beta ratio (beta frequency power inside the DLOR: beta frequency power outside the STN) was significantly higher in the Parkinson's disease group. Mann–Whitney *U* test, *P* < 0.001, *U* = 441.0, *n* = 31 for essential tremor, *n* = 62 for Parkinson's disease. (**D**) The mean beta frequency power was significantly higher in the Parkinson's disease group. Mann–Whitney *U* test, *P* < 0.001, *U* = 484.0, *n* = 31 for essential tremor, *n* = 62 for Parkinson's disease. (**E**) The mean power for other frequency ranges. Mann–Whitney *U* tests (*n* = 31 for essential tremor, *n* = 62 for PD) revealed a significant difference in gamma power between groups (*U* = 491.0, *P* < 0.001), which remained significant after Bonferroni correction (*α* = 0.0125). Alpha band differences (*U* = 627.0, *P* = 0.025) did not survive correction. Theta and delta band comparisons were not significant. Parkinson's disease group is shown in blue and essential tremor group is shown in red. For **C–E**, dots represent individual trajectories, white box represents the interquartile range, solid horizontal bar within the white box shows the median, whiskers show minimum and maximum and width of the violin shows density of data points. Created in BioRender. [Halen Erdman]. (2025) https://app.biorender.com/illustrations/668a490f2c61e64fe1f7901d.

Spectral analysis was performed to investigate frequency band power differences. A visual analysis of group-averaged spectrograms clearly shows an increase in the beta frequency band power in the Parkinson’s disease group but this signature is glaringly absent in the essential tremor group (Fig. 4A and B; Supplementary Fig. 1). This difference was quantified in both absolute and relative terms. The absolute mean beta frequency power within the motor subregion of the STN for each group was calculated, compared and found to be significantly different (Fig. 4D, *P* = 0.0005). Next, for each trajectory, the beta frequency band power within the motor subregion was compared with the beta frequency band power before STN entrance to produce the beta ratio. When the beta ratio of the Parkinson's disease group was compared with that of the essential tremor group, a significant difference was also found (Fig. 4C, *P* < 0.001). Delta, theta, alpha and gamma frequency band power was likewise compared between groups. Delta and theta frequency band powers did not differ (*P* = 0.95, *P* = 0.54, respectively), but alpha (*P* = 0.032) and gamma (*P* = 0.001) frequency band powers were increased in the Parkinson’s disease group (Fig. 4E). To investigate whether there was an effect of the symptom profile, a Parkinson’s disease subgroup consisting of patients who were designated as mixed tremor-rigid was compared with the essential tremor group (Supplementary Fig. 2). Both the beta ratio and mean beta power remained significantly elevated in the mixed symptom Parkinson’s disease group (*P* < 0.0001 and *P* < 0.0001, respectively).

### Regional spiking activity and beta frequency power patterns are conserved in *y*-coordinate matched groups

**Figure 5:**
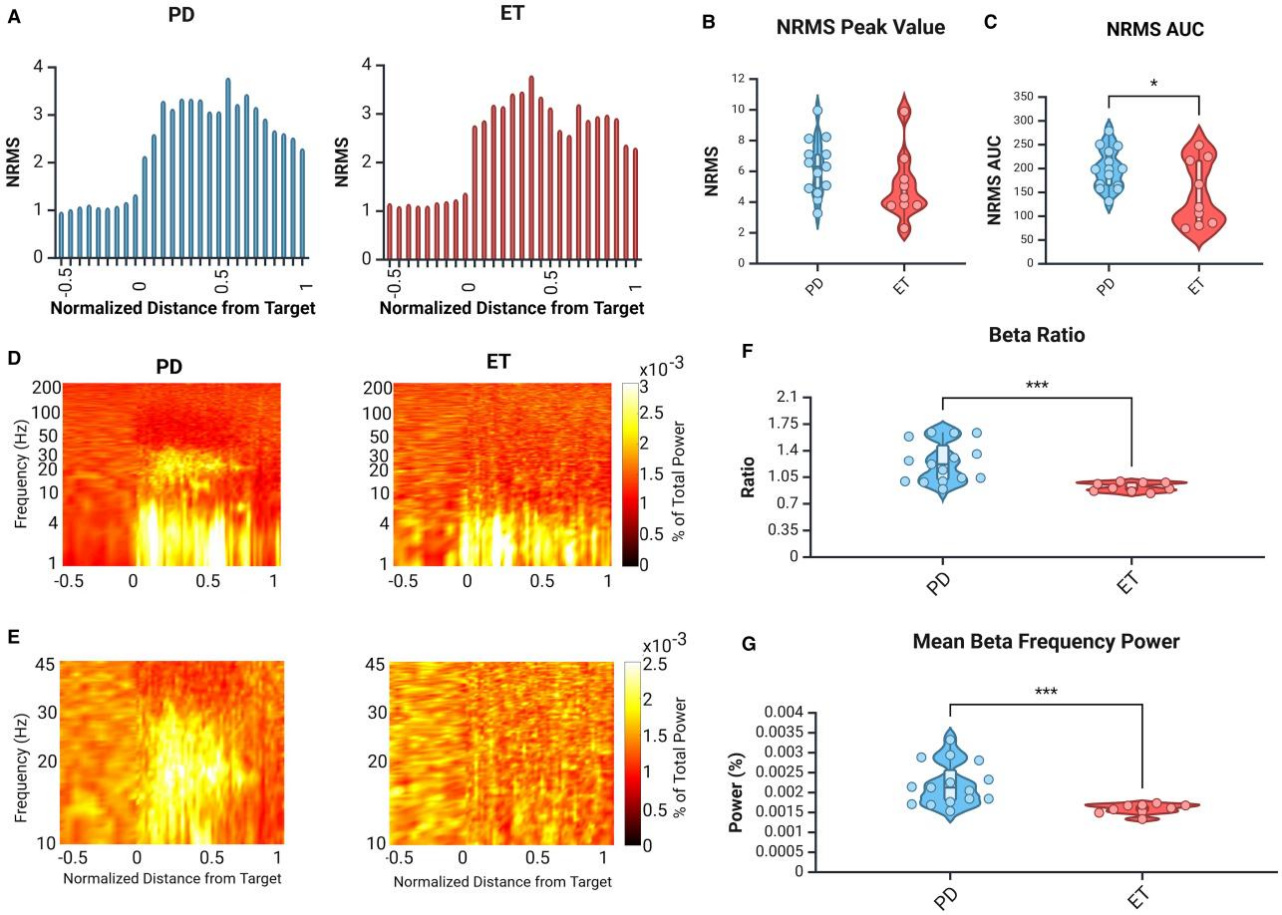
**Regional spiking activity and frequency distribution changes are upheld when patients are y-coordinate matched.** (**A**) Group-averaged NRMS analysis along the trajectory in the *y*-matched Parkinson's disease group (left, blue) and *y*-matched essential tremor group (right, red). Zero represents the entrance to the STN and one represents the exit. (**B**) Peak NRMS values were not significantly different between *y*-matched groups. Unpaired *t*-test, *P* = 0.18, *t* = 3.77, *n* = 10 for essential tremor, *n* = 15 for Parkinson's disease. (**C**) AUC of the NRMS was increased in *y*-matched Parkinson's disease patients. Unpaired *t*-test, *P* = 0.035, *t* = 2.26, *n* = 10 for essential tremor, *n* = 15 for Parkinson's disease. (**D**) Group-averaged spectrograms for the *y*-matched Parkinson's disease group (left) and *y*-matched essential tremor group (right) for a frequency range of 1–200 Hz. (**E**) Recalculated and zoomed-in group-averaged spectrograms for the *y*-matched Parkinson's disease group (left) and *y*-matched essential tremor group (right) to better see the beta band (13–30 Hz) for a frequency range of 10–45 Hz. (**F**) The beta ratio was significantly higher in the *y*-matched Parkinson's disease group. Mann–Whitney *U* test, *P* < 0.001, *U* = 7.0, *n* = 10 for essential tremor, *n* = 15 for Parkinson's disease. (**G**) The mean beta frequency power was significantly higher in the *y*-matched Parkinson's disease group. Mann–Whitney *U* test, *P* < 0.001, *U* = 8.0, *n* = 10 for essential tremor, *n* = 15 for Parkinson's disease. *y*-matched Parkinson's disease group is shown in blue and *y*-matched essential tremor group is shown in red. For **B**, **C**, **F** and **G**, dots represent individual trajectories, white box represents the interquartile range, solid horizontal bar within the white box shows the median, whiskers show minimum and maximum and width of the violin shows density of data points. Created in BioRender. [Halen Erdman]. (2025) https://app.biorender.com/illustrations/668533c558879a0a944087ea.

To overcome potential differences in the *y*-coordinate location between the groups, patients who shared relatively similar *y*-coordinates were gathered and compared in the same manner as the full groups. Again, the overall regional spiking activity was decreased in the STN of this subset of essential tremor patients compared to *y*-coordinate matched Parkinson's disease patients (Fig. 5A) as quantified by the AUC (Fig. 5C, *P* = 0.03). However, the peak NRMS values did not differ significantly (Fig. 5B, *P* = 0.18). Similar to the full group comparison, spectral analysis revealed an increase in the beta frequency band power in the DLOR of Parkinson's disease patients, but not in *y*-coordinate matched essential tremor patients (Fig. 5D and E). Both the beta ratio and mean beta frequency band power within the STN significantly differed (Fig. 5F, *P* = 0.0016; Fig. 5G, *P* = 0.003). When other frequency ranges were compared, only gamma frequency band power remained significantly different (*P* = 0.004).

### Beta frequency power increase remains in posterior subthalamic nucleus of Parkinson’s disease patients

**Figure 6:**
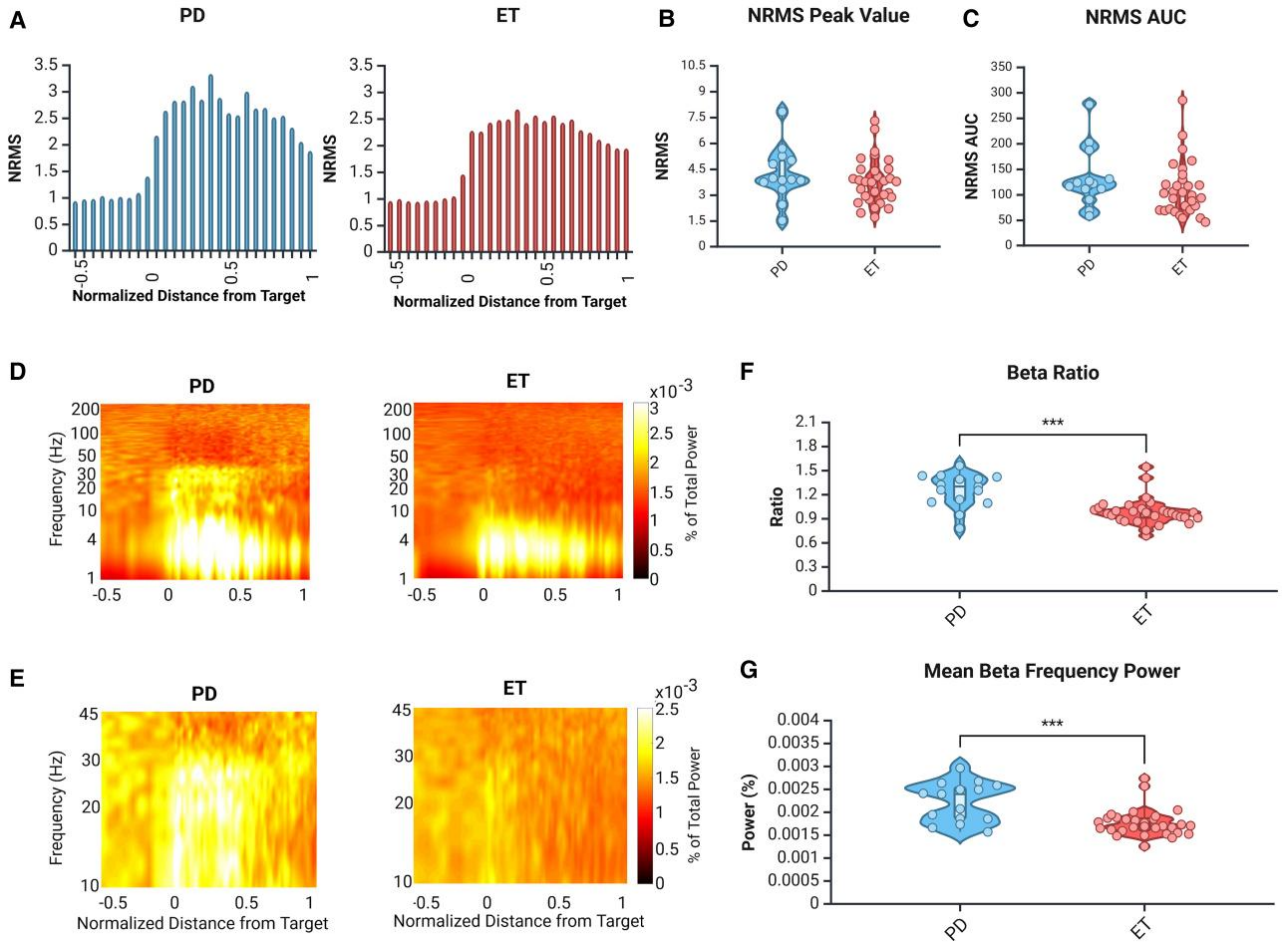
**Regional spiking activity does not differ but frequency distribution changes are upheld in Parkinson's disease posterior STN comparison.** (**A**) Group-averaged NRMS analysis along the trajectory in the posterior Parkinson's disease group (left, blue) and full essential tremor group (right, red). Zero represents the entrance to the STN and one represents the exit. (**B**) Peak NRMS values were not significantly different between groups. Unpaired *t*-test, *P* = 0.34, *t* = 0.96, *n* = 31 for essential tremor, *n* = 15 for Parkinson's disease. (**C**) AUC was not significantly different between groups. Unpaired *t*-test, *P* = 0.23, *t* = 1.23, *n* = 31 for essential tremor, *n* = 15 for Parkinson's disease. (**D**) Group-averaged spectrograms for the posterior Parkinson's disease group (left) and full essential tremor group (right) for a frequency range of 1–200 Hz. (**E**) Recalculated and zoomed-in group-averaged spectrograms for the posterior Parkinson's disease group (left) and full essential tremor group (right) to better see the beta band (13–30 Hz) for a frequency range of 10–45 Hz. (**F**) The beta ratio was significantly higher in the posterior Parkinson's disease group. Mann–Whitney *U* test, *P* < 0.001, *U* = 70.0, *n* = 31 for essential tremor, *n* = 15 for Parkinson's disease. (**G**) The mean beta frequency power was significantly higher in the posterior Parkinson's disease group. Mann–Whitney *U* test, *P* < 0.001, *U* = 89.0, *n* = 31 for essential tremor, *n* = 15 for Parkinson's disease. Posterior Parkinson's disease group is shown in blue and essential tremor group is shown in red. For **B**, **C**, **F** and **G**, dots represent individual trajectories, white box represents the interquartile range, solid horizontal bar within the white box shows the median, whiskers show minimum and maximum and width of the violin shows density of data points. Created in BioRender. [Halen Erdman]. (2025) https://app.biorender.com/illustrations/668cf9160c0e7012c5dd01bb.

To further overcome potential location differences within the STN that might contribute to firing or frequency distribution changes between groups, we selected a group of Parkinson's disease patients that showed clear electrophysiology of the STN in the electrode located in the posterior BenGun position (2 mm posterior to the central position, the planned PD-STN target) and compared them to the full essential tremor patient group. Regional spiking activity within the STN did not differ between the posterior STN Parkinson's disease group and the essential tremor group (Fig. 6A) as quantified by the peak or AUC of the NRMS (Fig. 6B, *P* = 0.34; Fig. 6C, *P* = 0.23). Nonetheless, in the spectral analysis, the beta frequency power is markedly higher in the posterior STN Parkinson's disease group compared to the essential tremor group. This is evident from visual inspection of the spectrograms (Fig. 6D and E) and quantified by statistical differences in both the beta ratio and mean beta frequency power (Fig. 6F, *P* < 0.0001; Fig. 6G, *P* < 0.001). As in the *y*-coordinate matched comparison, gamma frequency band power was the only other frequency band to remain significantly different between the essential tremor and the Parkinson's disease groups (*P* < 0.001).

## Discussion

The findings of this study suggest that beta frequency oscillations in the STN are distinctly increased in Parkinson’s disease compared to essential tremor. This finding was upheld even after correcting for potential location differences within the STN. Additionally, the regional spiking activity of the STN seems to be increased in Parkinson's disease patients, potentially reflecting its overactive state in this disease.

Abnormal oscillatory activity plays a pivotal role in the manifestation of motor symptoms in both ET^25-27^ and Parkinson's disease,^14-16^ albeit through different mechanisms. Tremor, though action tremor in essential tremor and resting tremor in Parkinson's disease, is a common feature between the two diseases. In essential tremor, the tremor is primarily associated with abnormal rhythmic firing in the tremor frequency (6–12 Hz) within the cerebello-thalamo-cortical circuit.^28^ This pathological oscillatory activity is characterized by excessive synchronization of neuronal firing in the cerebellum and thalamus, which manifests as the kinetic and postural tremors characteristic of essential tremor.^28-30^ Conversely, in Parkinson's disease, the tremor is linked to disrupted oscillatory activity in the basal ganglia-thalamo-cortical circuits due to dopaminergic neuron degeneration in the substantia nigra pars compacta.^31,32^ However, the cerebral networks probably play a major role in the maintenance and augmentation of Parkinson's disease tremor phenomena.^33,34^ Cells that fire in synchrony with a patients tremor, so called tremor cells, can be observed in the STN.^35^ This can then sometimes be seen as an increase in the power at the tremor frequency range in the spectral analysis. However, we did not find a significant difference in the neuronal activity at the essential tremor tremor frequency range when we compared the essential tremor and Parkinson's disease groups. Nevertheless, this can potentially be explained by studies showing that although tremor-related activity can be seen in the basal ganglia of Parkinson's disease patients and animal models, usually it is less common than expected and notably asynchronized.^22,36^

The results presented here bring into question whether beta oscillations are a feature of the healthy STN, if they are truly pathological and specific to Parkinson's disease, or if perhaps the lack of beta oscillations in the essential tremor STN is an active process and is a pathological characteristic of this disease. There have been studies that suggested that beta oscillations are present and necessary for normal motor movement.^37,38^ Further, some suggest the level of synchronization of STN activity in the beta band may play a crucial role in determining whether motor programming and movement initiation are facilitated or inhibited.^39^

Parkinson's disease dopaminergic degeneration leads to robust increased beta band oscillations (13–30 Hz) in the basal ganglia, which are transmitted to the neuronal targets of the basal ganglia, most probably subserving as an augmented stop signal and contributing to the akinesia/bradykinesia and rigidity Parkinson's disease symptoms.^14,15^ Increased beta oscillations reflect abnormal synchronization of neural firing, which probably impedes the flexibility of motor control, making movements slow and rigid. DBS targeting the STN or GPi can reduce beta oscillations, thereby alleviating the motor symptoms in Parkinson's disease.^40^ Though common, abnormal beta oscillations are not a universal feature in Parkinson's disease due to several factors. Variability in disease progression and individual differences in brain structure and function can influence their presence. Dopaminergic medications, such as levodopa, can reduce these oscillations, making them less detectable in medicated patients.^40^ Additionally, exact recording location and methods, symptom heterogeneity and the presence of coexisting neurological conditions can affect the observation of beta oscillations. Our results agree with the majority of literature suggesting that there are increased beta oscillations in the motor domain of the STN of Parkinson's disease patients and that they are a pathological feature. However, more research needs to be done to investigate individual differences in beta frequency power to better understand the mechanism of its pathology in depth. In addition to increases in beta frequency power, broad-band gamma frequency power was also increased in all comparisons in the Parkinson's disease group. This agrees with reports of increased cortical gamma power when comparing Parkinson's disease and essential tremor patients^41^ and makes sense given the connectivity between the basal ganglia and the cortex.

The relationship between beta oscillations and essential tremor remains even less clear. While there are reports of the presence of beta oscillatory disturbances in essential tremor, these findings are not as consistent or pronounced as those observed in Parkinson's disease.^42-45^ The essential tremor tremor is generally characterized by different frequency bands than the Parkinson's disease tremor, often in the 6–12 Hz range for essential tremor and 4–7 Hz for Parkinson's disease resting tremor,^46^ implicating the cerebellum and thalamus more directly.^26,47^ However, the lack of beta oscillations in our essential tremor group presented here could also be interpreted as the absence of the stop signal that is overactive in Parkinson's disease, causing excessive movement in the form of tremor. In fact, Asch *et al.*^48^ investigated the connection between tremor frequency power and beta frequency power in Parkinson's disease patients and posited that they have an inverse relationship. The exact role of beta oscillations in essential tremor requires further investigation, as their presence and significance in essential tremor have not been definitively established. However, given the anatomical and functional connectivity between the cerebellum, thalamus and STN,^49^ it is plausible that cerebellar-driven tremor oscillations do not entrain the STN in essential tremor, preserving normal beta band activity.

The neurotransmitter dynamics within these circuits may further explain the differential beta activity observed in Parkinson's disease and essential tremor. Beta oscillations in the STN are modulated by the interplay between excitatory glutamatergic input from the motor cortex and inhibitory GABAergic signalling from the external globus pallidus (GPe).^50^ In Parkinson's disease, dopamine depletion disrupts this balance, reducing GABAergic inhibition and leading to excessive STN activity and beta synchronization.^51^ Additionally, the hyperactive STN provides excessive glutamatergic output to the internal globus pallidus (GPi) and substantia nigra pars reticulata (SNr), further reinforcing beta band pathological oscillations.^52^ In contrast, essential tremor is driven by excessive cerebellar excitatory output to the thalamus, which does not inherently alter basal ganglia beta synchronization. The cerebellum’s glutamatergic drive to the thalamus in essential tremor may facilitate low-frequency tremor oscillations, but without significant disruption of GABAergic regulation in the basal ganglia, the STN may not develop the pathological beta synchronization seen in Parkinson's disease.

These findings align with the ‘dimmer switch’ model, which suggests that tremor emerges from interactions between two distinct but interacting systems: the cerebello-thalamo-cortical loop, which acts as a ‘tremor amplifier,’ and the basal ganglia, which functions as a ‘dimmer switch’ that normally suppresses tremor.^33^ In Parkinson's disease, impaired basal ganglia function fails to regulate tremor, allowing pathological oscillations to manifest. In essential tremor, however, the basal ganglia’s dimmer switch remains intact, and tremor persists due to intrinsic oscillations within the cerebellar network rather than a loss of inhibitory control.

Current treatment for essential tremor and Parkinson’s disease typically begins with medications and other non-invasive treatments. If these treatments become ineffective or cause intolerable side effects, DBS may be considered as an advanced therapeutic option. While DBS targeting the STN has been consistently used for many years with good results for Parkinson's disease,^53^ optimal DBS targets for essential tremor are still being explored. The PSA has emerged as a promising target for DBS in the treatment of essential tremor. Located near the junction of the STN and the zona incerta, the PSA plays a crucial role in modulating motor function and has been found to be highly effective in reducing tremor severity.^12,54-56^ Clinical studies have demonstrated that DBS of the PSA results in substantial improvements in tremor control, often with fewer side effects compared to other DBS targets like the VIM of the thalamus.^11,12,54-56^ As a result, DBS targeting the PSA offers a viable and effective alternative for patients with essential tremor, particularly those who may not respond adequately to other treatments. The dual electrode technique used in this study, which involves harnessing the clear electrophysiological signal of the STN to detect the electrophysiologically quiet PSA, may increase precision to this target and, therefore, increase effectiveness for patients.

By uncovering distinct oscillatory dynamics in essential tremor and Parkinson's disease, the most common movement disorders, our findings can deepen our understanding of the neural mechanisms underlying motor symptoms and to inform targeted therapeutic interventions through DBS. However, this study’s conclusions are limited. The retrospective design relies on previously collected data and although the electrophysiological team was consistent, the data came from two medical centres which have minor differences in procedure and planning. While efforts were made to match *y*-coordinates between groups, differences in trajectory angles and positioning during surgery may still lead to inherent variability in the recordings. There may also be other confounding variables that could impact neurophysiological outcomes, such as comorbidities, medication history, age, sex or disease duration and severity, that were not extensively controlled or analysed here. Specifically, including more tremor dominant Parkinson's disease patients could be beneficial. Despite these limitations, we believe these findings contribute to advancing the field’s knowledge of the pathophysiology of Parkinson's disease and essential tremor and provide insights into potential biomarkers and treatment strategies for these debilitating conditions.

## Supplementary Material

fcaf297_Supplementary_Data

## Data Availability

Anonymized data will be made available upon request from the corresponding author. Custom Matlab 2018b codes developed for this manuscript are available at https://github.com/halenerdman/Beta-ET-PD.
