# Supplementary material for: Pathological beta power increase in the subthalamic nucleus is absent in essential tremor
Source: Brain Commun. 2025 Aug 14;7(5):fcaf297. doi: 10.1093/braincomms/fcaf297 (PMC12402545; doi:10.1093/braincomms/fcaf297)
Supplement: fcaf297_Supplementary_Data [file fcaf297_supplementary_data.pdf]

Supplementary Materials

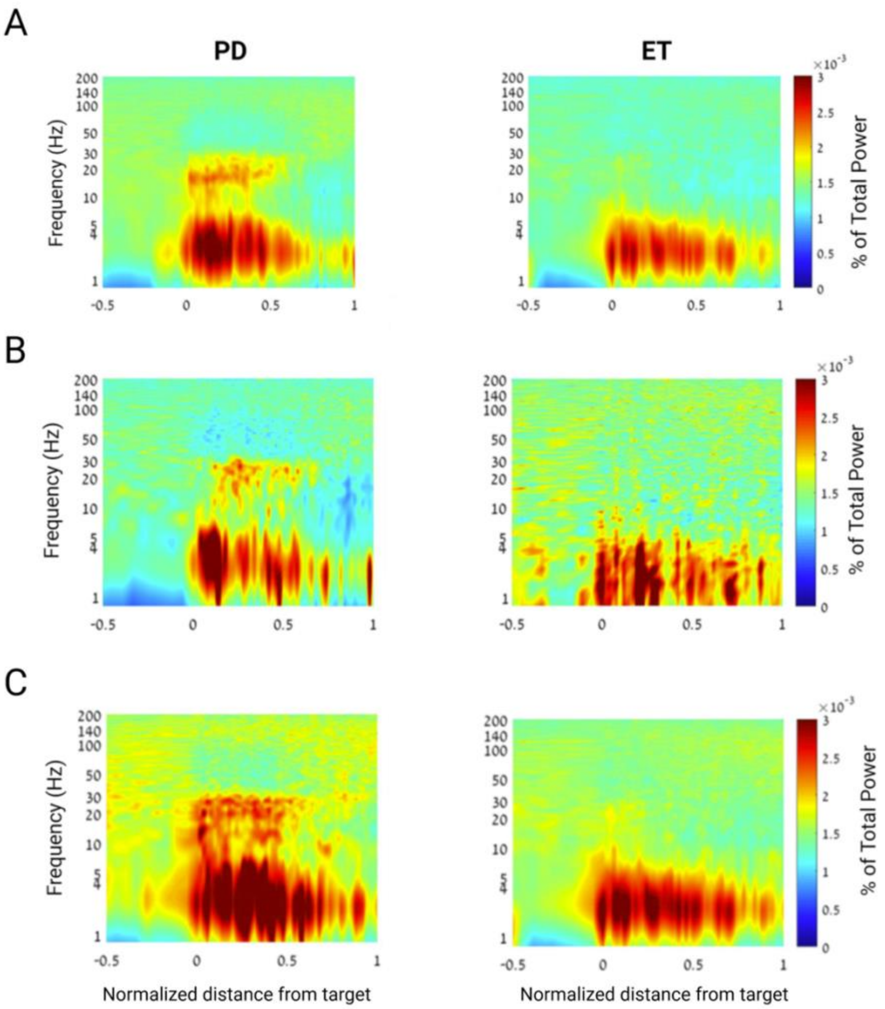

Supplementary Figure 1. Beta band power is increased in PD. A. Group averaged spectrograms for the PD group (left) and ET group (right) for a frequency range of 1-200 Hz (same data as presented in figure 4A) using standard heatmap colors. B. Group averaged spectrograms for the y-matched PD group (left) and y-matched ET group (right) for a frequency range of 1-200 Hz (same data as presented in figure 5D) using standard heatmap colors. C. Group averaged spectrograms for the posterior PD group (left) and full ET group (right) for a frequency range of 1-200 Hz (same data as presented in figure 6D) using standard heatmap colors. Figure generated with BioRender available at https://app.biorender.com/illustrations/67b6e8725a3c1b221bbd5fef

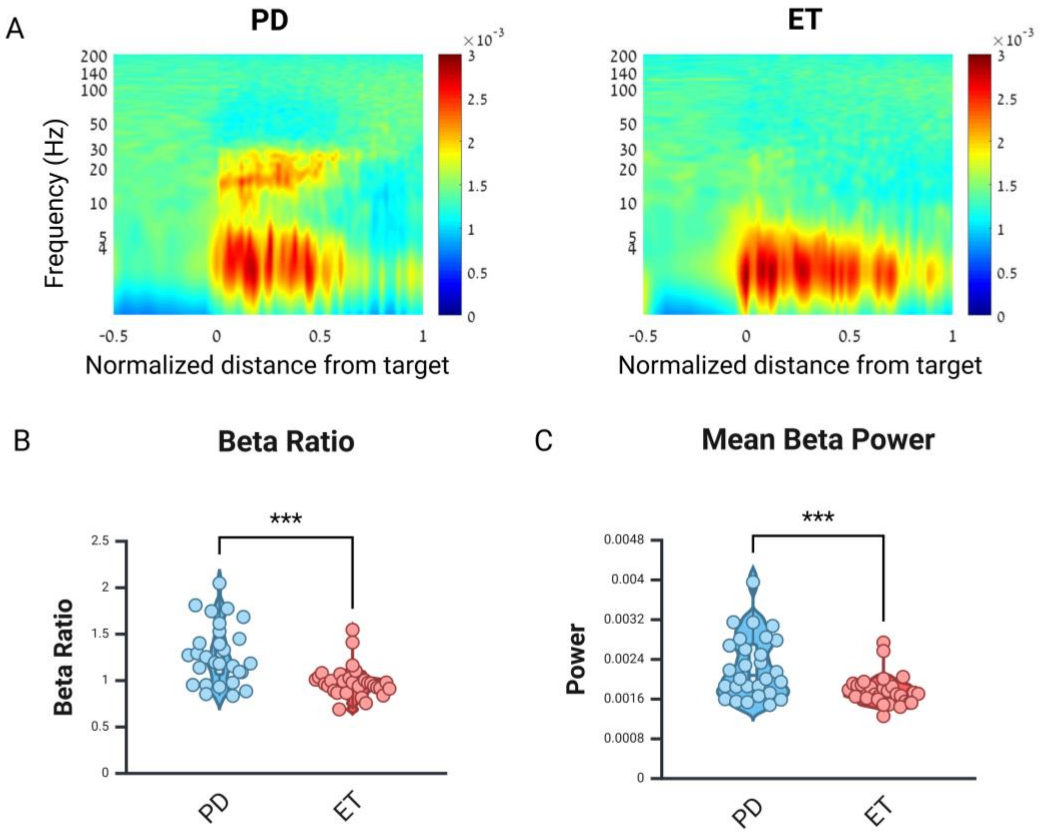

Supplementary Figure 2. A mixed tremor-rigidity symptom PD patient group maintains an increase in beta band power. A. Group averaged spectrograms for the PD patients who had a mixed tremor-rigidity symptom profile (n=28 trajectories) and the full ET group for a frequency range of 1-200 Hz. B. The beta ratio was significantly higher in the mixed symptom PD group compared to the ET group. Mann-Whitney U test, $p<0.001$, $U=179.0$, $n=31$ for ET, $n=28$ for PD. C. The mean beta power was significantly higher in the mixed symptom PD group compared to the ET group. Mann-Whitney U test, $p<0.001$, $U=209.0$, $n=31$ for ET, $n=28$ for PD. Dots represent individual trajectories, white box represents the interquartile range, solid horizontal bar within the white box shows the median, whiskers show minimum and maximum, width of the violin shows density of data points. Figure generated using BioRender available at https://app.biorender.com/illustrations/67bc5be6c4243ef38bf4a86e
